# Comparative Assessment of Retention and Caries Protective Effectiveness of a Hydrophilic and a Conventional Sealant—A Clinical Trial

**DOI:** 10.3390/children9050646

**Published:** 2022-04-30

**Authors:** Liana Beresescu, Mariana Pacurar, Alexandru Vlasa, Alexandra Mihaela Stoica, Timea Dako, Blanka Petcu, Daniela Eșian

**Affiliations:** Faculty of Dental Medicine, George Emil Palade University of Medicine, Pharmacy, Science, and Technology of Targu Mures, 540139 Târgu-Mureș, Romania; liana.beresescu@umfst.ro (L.B.); mariana.pacurar@umfst.ro (M.P.); timea.dako@umfst.ro (T.D.); blanka.petcu@umfst.ro (B.P.); daniela.esian@umfst.ro (D.E.)

**Keywords:** moisture-tolerant resin-based sealant, conventional resin-based sealant, retention, dental decay

## Abstract

Sealants are highly efficient and the most secure method for the prevention of caries lesions from pits and fissures in recently erupted permanent teeth. The aim of this study is to clinically assess and compare the retention and evolution of caries of a moisture-tolerant resin-based sealant with a conventional hydrophobic resin-based sealant. Material and method: We have included in the study 28 children with between 6 and 8 years old. For each child we sealed 4 permanent molars (a total of 112 teeth). The study group was divided into two subgroups: the Embrace Group—consisting of 56 first permanent molars that underwent dental sealing with moisture-tolerant resin-based fissure sealant (Embrace™ WetBond™ Pulpdent, Watertown, MA, USA) and the Helioseal Group—represented by the same number of 56 first permanent molars that were sealed with conventional hydrophobic resin-based sealant (Helioseal F™, Ivoclar Vivadent Schaan, Liechtenstein). The retention and the incidence of new carious lesions of each sealant were assessed clinically at 6, 12, 18, and 24 months. Results: The 12-month follow-up assessment showed perfect integrity in 50 molars (89.28%) sealed with moisture-tolerant resin-based material (Embrace Group), and in 51 molars (91.07%) with conventional resin-based sealant (Helioseal Group). At the 24-month recall, the retention was maintained in 44 molars (78.57%) in the Embrace Group and in 45 molars (80.35%) in the Helioseal Group, respectively. The follow-up assessments showed no statistically significant differences (*p* > 0.5) between the two materials regarding sealant retention. First evidence of new carious lesions was present at 12 months on two molars sealed with Embrace WetBond and on one molar sealed with Helioseal. At the 24-month evaluation, the prevalence of caries in the Embrace Group was 7.14% (four caries) and 3.56% (two caries) in the Helioseal Group. Moreover, there were no statistically significant differences (*p* > 0.05) between the two materials regarding new caries development at any of the follow-up assessments. Conclusions: Moisture-tolerant resin-based sealant was effective in terms of retention and caries prevention.

## 1. Introduction

Over the last several years, dental caries prevalence among children and teenagers has suffered a slight decline [1]. Nevertheless, caries lesions still represent an important health problem worldwide [2] affecting 60–90% of children [3,4,5,6,7,8]. Most caries affecting young teeth start by destroying occlusal surfaces. Pit and fissures of the new erupted permanent molars represent the ideal place for caries lesion development due to their susceptibility to harboring dental plaque [9,10]. Because dental caries evolution involves the interaction of a complex series of factors such as a large number of bacteria, host resistance, diet, time, and other factors that vary among individuals, it is difficult to predict the teeth that will be affected [11,12].

From professional procedures for preventing dental caries, fluorides are very efficient in reducing the incidence of carious lesions that occur on the smooth surfaces of teeth. Unfortunately, fluorides are not equally effective in protecting occlusal pits and fissures [2,13]. Applying a sealant proved to be a highly effective means of preventing carious lesion in this case [14,15]. The efficiency of pit and fissure sealing for dental caries prevention has often been reported [16,17,18,19,20]. Current recommendations state that sealing pits and fissures of permanent molars and premolars is efficient and useful in maintaining healthy dental tissues; therefore, dentists should be guided to use sealants [21].

A material used for sealing pits and fissures, areas that that are the most frequent affected by caries, is called a fissure sealant. It creates a hindrance that interrupts the nutrition source of the biofilm, and as a result, it reduces the development of cariogenic bacteria [9,22]. It is well known that pits and fissures represent zones with a high deposit of mutans streptococci. Sealing these zones leads to a significant reduction of the bacteria on the treated tooth surface and also reduces their oral count [23]. There is an intense correlation between these retentive zones, the sealing material, and the incidence of caries lesions. If the sealant remains in perfect condition, the carious process will not be able to grow underneath [24,25]. That is why the retention of this material is the primary condition for the success of sealing. To increase the resistance of pit and fissure sealant materials, several materials and techniques have been proposed [26]. Today, resin-based and glass ionomer-based pit and fissure sealants are widely used [27]. Several published studies [28,29] proved that composite resins are currently the material of choice for sealants because of their higher retention compared to glass ionomer cements. Yet, resin-based materials have the disadvantage of being hydrophobic and technique-sensitive, which require increased control of the saliva level. In certain situations, such as with uncooperative patients or those with disabilities, salivary control is very challenging and difficult [30].

Currently, resin-based sealants are undergoing increased and alert development due to the improvement of moisture-tolerant chemistry. For example, Embrace™ WetBond™ (Pulpdent, Watertown, MA, USA) is a sealant containing resin, no BisGMA, and no Bisphenol A, and it uses hydrophilic resin chemistry. Embrace incorporates di-tri and multifunctional acrylate monomers into an advanced acid-integrating chemistry that is activated by moisture [31]. Due to its chemical composition, it acts effectively in a humid environment, has moisture adhesion, is a less sensitive technique, has better retention, superior marginal sealing, and increased fluoride release. As a result, so, the sealant could be a proper aid in cases in which an absolute isolation is difficult [32,33].

The aim of this study is the clinical assessment of retention and evolution of caries when sealed with moisture-tolerant resin-based sealant compared to a conventional hydrophobic resin-based sealant over a period of 24 months.

## 2. Materials and Methods

The study methodology was approved by the ethics committee of the Denta Aur Private Medical Center, Tîrgu Mureș, Romania, with the clinical trial registration number 011/04.01.2019.

Written informed consent was obtained from the parents of all participating children. 

Two calibrated dentists performed all the clinical steps. They were helped by trained chairside dental assistants. Before the examinations, operators completed an ICDAS-II calibration course, assessing the condition of tooth surfaces and the presence of caries according to the International Caries Detection and Assessment System (ICDAS-II). They also had the opportunity to become familiar with the application techniques of the two materials.

For the clinical examination of molars, we used two examination methods: visual and tactile. For the visual examination, we cleaned and dried the teeth using a dental unit air/water spray, and for the tactile examination, we used a rounded tip dental probe to clean the plaque and food debris from the pits and fissures [34]. 

The inclusion criteria were as follows: the presence of all four completely erupted permanent first molars without dental abnormalities and with deep pits and fissures susceptible to tooth decay and no clinical signs of caries lesions. If the integrity of the tooth was doubtful, the suspicious teeth/children were excluded from the study. Teeth/children with fillings and sealants were also excluded. 

**Figure 1:**
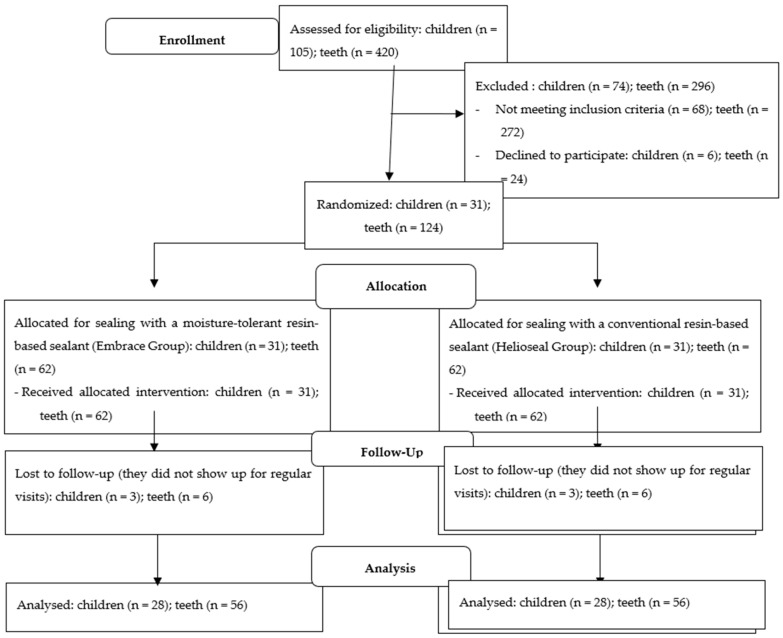
Consort flow diagram for the study.

In order to be recruited in our study, we examined 105 children (420 teeth) aged between 6 and 8 years. we excluded 74 children (296 teeth), because 68 children (272 teeth) did not meet the inclusion criteria (they had carious lesions, filings, or sealants), and 6 children or parents (24 teeth) declined to participate. Only 31 children had the indication for sealing for all their first permanent molars (124 teeth). Wishing to have as few variables as possible, we decided to base our study on these children’s molars. For 3 children, we could not perform all the periodic checks, so we had to exclude them throughout the study period. A total of 28 healthy cooperative children who were at caries risk with all four recently newly erupted permanent first molars (112 teeth) were included in the study (Figure 1). 

The children included in the study had not benefited from systemic fluoridation and had good oral hygiene. In addition, all patients received instructions about good oral hygiene and diet.

The required sample size was determined to be 112 teeth (56 teeth per group) using G-power software™ Heinrich Heine University, Dusseldorf, Germany, for Windows, for a power of 95% (α = 0.05, β = 0.05).

There were 112 first permanent molars that underwent a preventive dental sealing procedure. We chose to use the split-mouth design method [35]. According to this method, an equal number of fissure sealants were applied to the maxillary and mandibular teeth and to the left and right side of both studied materials. Depending on the type of material we used, teeth were divided into two subgroups:

- Embrace Group = 56 teeth sealed with a moisture-tolerant resin-based sealant Embrace™ WetBond™ Pulpdent, Watertown, MA, USA.
- Helioseal Group = 56 teeth sealed with a conventional resin-based sealant Helioseal F™, Ivoclar Vivadent Schaan, Liechtenstein

The application of the sealant was performed according to the manufacturer’s instructions.

For conventional resin-based sealant, the steps were: tooth cleaning with pumice without fluoride, rinsing with water and air-drying, cotton isolation of the tooth, air-drying of the tooth, application of the phosphoric acid gel (37%) for 30 s, rinsing and air-drying, control of the acid-etched dental surface, bonding application, light curing of the bonding, sealant application, light curing of the sealant, control of marginal adaptation and occlusion. 

For moisture-tolerant resin-based sealant we followed the same steps with two exceptions: thorough air-drying after the enamel etching and bonding. In this case, after rinsing, the teeth were lightly dried using a cotton roll but not desiccated. We kept the occlusal surface of the teeth slightly moist, so it was glossy and shiny prior to the application of the sealant material. 

Evaluation of sealant retention and development of caries was performed at 6, 12, 18, and 24 months. At every follow-up assessment, we evaluated the integrity and marginal adaptation of the sealant through visual and tactile examination.

For assessing sealant retention, we used Simonsen’s criteria [36]:

- I: Totally retained sealant.
- II: Partially retained sealant.
- III: Missing sealant.

### Statistical Analysis

For evaluation of the categorical data, we used Fisher’s exact test and chi-squared test. The chosen significance level was set at 0.05. and p was considered significant when *p* ≤ 0.05. All data were recorded using GraphPad Prism™ V6.01 software for Windows™ 2017.

## 3. Results

A total of 112 teeth were sealed in 28 patients, and all patients attended all follow-up intervals, resulting in a recall rate of 100%. 

The 6-, 12-, 18-, and 24-month follow-ups showed no statistically significant differences (*p* > 0.05) between the two sealing materials regarding sealant retention (Table 1, Table 2, Table 3 and Table 4). 

At the 6-month follow-up interval the Embrace Group the Helioseal Group had similar percentages of completely retained (94.64%, 96.42%), partially retained (1.78%, 0) and missing (3.57%, 3.57%) sealants, respectively. 

At the 12-month follow-up, approximately 90% of the sealants in both groups were totally retained, almost 4% were partially retained, and approximately 6% were missing.

At the 18-month follow-up evaluation, 85.71% of sealants were totally retained in the Embrace Group and 89.28% in the Helioseal Group; 5.35% were partially retained in the Embrace Group and 1.78% in the Helioseal Group. A percentage of 8.92% from both groups of sealants was missing.

At the end of our trial, at 24 months, in the Embrace Group 78.57% of sealants remained completely retained, whereas in the Helioseal Group 80.35% were retained; 8.92% and 10.71%, respectively, from the Embrace and Helioseal Groups were partially retained. From the Embrace Group, 12.5% of sealants were missing, and from the Helioseal Group 8.92% were missing.

The 6-, 12-, 18-, and 24-month follow-up assessments also showed no statistically significant differences (*p* > 0.05) between the two sealing materials concerning new carious lesion development (Table 5, Table 6, Table 7 and Table 8). 

At the 6-month follow-up, there were no carious lesions in either group. Evidence of new caries lesion was present at 12 months: two molars sealed with Embrace WetBond, and one molar sealed with Helioseal. At the 18-month follow-up, three molars from the Embrace Group and two molars from the Helioseal Group showed dental decay at the end of 24 months, the percentage of caries lesions in the Embrace Group was 8.92% (five caries) and 5.35% (three caries) in the Helioseal Group.

## 4. Discussion

The present study compared the clinical performance of a hydrophilic resin-based fissure sealant with a conventional hydrophobic resin-based sealant over a period of 24 months. In terms of retention or incidence of new carious lesions formation, there was no statistically significant difference between the two sealing materials. Both used fissure sealants showed a similar efficiency in preventing dental caries on the sealed surfaces. This is due to their good retention and fluoride release potential [37]. The addition of fluoride to sealants was based on the finding from more than 30 years ago that the prevalence of secondary caries was considerably reduced around fluoride-releasing materials such as ionomers used for restorations [38]. Fluoridated sealants have demonstrated antibacterial properties and a greater caries resistance compared with nonfluorinated ones [39,40]. It also has shown caries-inhibiting effects with an important reduction in the prevalence of wall caries and in the caries lesions depth [40]. This fact can be of great value because in practice, frequently, is difficult to put a correct diagnostic for a sound surface [41]. Another explanation of the effectiveness of sealants in preventing caries is that even in case of sealant loss, the rest of the material remains in the depth of pits and fissures and may have a protective role [42]. 

Our good results can also be correlated with the period we chose for sealing—as soon as it is possible after tooth eruption. This is the period recommended by the literature as the ideal time to seal a tooth because root development and all tooth hard structures mineralization continues in the years after eruption. During this period, tooth caries receptivity is maximum [43,44]. If the caries lesion does not occur in the years immediately after eruption, the probability for that tooth to become carious is very low. Sealing of adult teeth is not routinely recommended. Sealants should be placed on adult teeth if there is a high risk of caries as could occur in case of an excessive intake of sugar, xerostomia induced by radiation therapy, drugs, or other causes [45].

Our results are similar to other recent published studies [26,40,46,47], revealing the same effectiveness of the moisture-tolerant resin-based sealant compared to the conventional hydrophobic resin-based sealant. We have also found studies with results differing from ours, where the moisture-tolerant resin-based sealant showed higher retention and caries prevention effect than conventional resin-based sealant [48], or, in contrast, showed significantly lower retention and caries prevention of moisture-tolerant resin-based sealant than conventional resin-based sealant [49].

These contradictory findings highlight the need for more studies with longer follow-up periods to assess the performance of the newer hydrophilic sealants.

Moisture-tolerant resin-based materials could be an addition to pit and fissure sealants [27] because moisture contamination is a significant risk factor for material retention and resistance. In practice, we often have situations where it is difficult to attain good moisture control. The high possibility of occlusal caries development is during tooth eruption because the accumulation of dental plaque is more abundant on the occlusal surfaces of erupting teeth compared to teeth with proper occlusal contact [50]. As a result, the occlusal surfaces of recently erupted teeth are mainly affected by caries because of the reduced possibility of cleaning and the diminished natural cleaning mechanisms [51]. Moreover, newly erupted teeth are less mineralized because the post-eruptive maturation is not completed, and they have less resistance to acid attacks [37,52,53]. Thus, it is important to find a sealant with good clinical performance for use in partially erupted teeth, and for other cases, where proper isolation is challenging, such as in uncooperative patients or persons with psychiatric diagnoses [42,54]. 

This new generation of fissure sealants could be helpful in situations where maintaining isolation is difficult [32]. It also provides other advantages. Owning hydrophilic properties, it has lower technical sensitivity than conventional resin-based sealants [55]. It does not require a bonding agent, and it reduces the time needed for the procedure and increases patient cooperation [56]. A moisture-tolerant resin-based sealant, which is clinically efficient, could offer dentists a new alternative in pit and fissure sealing especially when moisture control is difficult.

### Limitations of the Study

It is mandatory to mention the limitations of the study. We have gathered and formed a specific group of children, and the follow-up period was short. This study is ongoing, and further results will be published.

## 5. Conclusions

Both resin-based sealant materials that were used in the study were effective regarding retention and preventions of caries lesions on young first permanent molars over a period of 24 months. Moisture-tolerant resin-based fissure sealant displayed a comparable retention rate as a conventional resin-based pit and fissure sealant. They are similarly effective in preventing caries development. 

## Figures and Tables

**Table 1:** Assessment of sealant retention rate after 6 months according to Simonsen’s criteria.

	I	II	III	Total	*p* = 0.603
Embrace	53 (94.64%)	1 (1.78%)	2 (3.57%)	56	
Helioseal	54 (96.42%)	0	2 (3.57%)	56	
Total	107 (95.53%)	1 (0.89)	4 (3.57%)	112 (100%)	

**Table 2:** Assessment of sealant retention after 12 months according to Simonsen’s criteria.

	I	II	III	Total	*p* = 0.5619
Embrace	50 (89.28%)	3 (5.35%)	3 (5.35%)	56	
Helioseal	51 (91.07%)	1 (1.78%)	4 (7.14%)	56	
Total	101 (90.17%)	4 (3.57%)	7 (6.25%)	112 (100%)	

**Table 3:** Assessment of sealant retention after 18 months according to Simonsen’s criteria.

	I	II	III	Total	*p* = 0.5943
Embrace	48 (85.71%)	3 (5.35%)	5 (8.92%)	56	
Helioseal	50 (89.28%)	1 (1.78%)	5 (8.92%)	56	
Total	98 (87.50%)	4 (3.57%)	10 (8.92%)	112 (100%)	

**Table 4:** Assessment of sealant retention after 24 months according to Simonsen’s criteria.

	I	II	III	Total	*p* = 0.8043
Embrace	44 (78.57%)	5 (8.92%)	7 (12.5%)	56	
Helioseal	45 (80.35%)	6 (10.71%)	5 (8.92%)	56	
Total	89 (79.46%)	11 (9.82%)	12 (10.71%)	112 (100%)	

**Table 5:** Assessment of carious lesion after 6 months.

Material	Yes	No	Total
Embrace	0	56	56
Helioseal	0	56	56
Total	0	112	112

**Table 6:** Assessment of carious lesion after 12 months.

Material	Yes	No	Total	*p* = 1.000
Embrace	2 (3.57%)	54 (96.42%)	56	
Helioseal	1 (1.78%)	55 (98.21%)	56	
Total	3 (2.67%)	109 (97.32%)	112	

**Table 7:** Assessment of carious lesion after 18 months.

Material	Yes	No	Total	*p* = 1.000
Embrace	3 (5.35%)	53 (94.64%)	56	
Helioseal	2 (3.57%)	54 (96.42%)	56	
Total	5 (4.46%)	107 (95.53%)	112	

**Table 8:** Assessment of carious lesion after 24 months.

Material	Yes	No	Total	*p* = 0.7163
Embrace	5 (8.92%)	51 (91.07%)	56	
Helioseal	3 (5.35%)	53 (94.64%)	56	
Total	8 (7.14%)	104 (92.85%)	112	

## Data Availability

Not Applicable.
